# SPARC and the N-propeptide of collagen I influence fibroblast proliferation and collagen assembly in the periodontal ligament

**DOI:** 10.1371/journal.pone.0173209

**Published:** 2017-02-28

**Authors:** Emilie Moore Rosset, Jessica Trombetta-eSilva, Glenn Hepfer, Hai Yao, Amy Dodd Bradshaw

**Affiliations:** 1 Department of Oral Health Sciences, Medical University of South Carolina, Charleston, South Carolina, United States of America; 2 Department of Bioengineering, Clemson University, Clemson, South Carolina, United States of America; 3 Gazes Cardiac Research Institute, Division of Cardiology, Department of Medicine, Medical University of South Carolina, Charleston, South Carolina, United States of America; 4 The Ralph H. Johnson Department of Veteran’s Affairs Medical Center, Charleston, South Carolina, United States of America; University of Bergen, NORWAY

## Abstract

The periodontal ligament (PDL) is a fibrous connective tissue that anchors tooth cementum into alveolar bone. Secreted protein acidic and rich in cysteine (SPARC) is a collagen-binding matricellular protein known to influence collagen fiber assembly in the PDL. In contrast, functional properties of the N-propeptide of collagen I, encoded in exon 2 of the COL1A1 gene, are poorly understood. In this study, the PDL of collagen I exon 2-deleted (wt/ko), SPARC-null (ko/wt), and double transgenic (ko/ko) mice were evaluated in terms of cellularity, collagen area, fiber morphology, and extraction force and compared to WT (wt/wt) mice. Picro sirius red staining indicated a decrease in total PDL collagen content in each of the transgenic mice compared to WT at 1 and 3 month age points. At 12 months, only SPARC-null (ko/wt) and double-null PDL demonstrated less total collagen versus WT. Likewise, an increase in thin PDL collagen fibers was observed at 1 and 3 months in each transgenic, with increases only in SPARC-null and double-null mice at 12 months. The force required for tooth extraction was significantly reduced in SPARC-null versus exon 2-deleted and WT mice, whereas double-null mice demonstrated further decreases in force required for tooth extraction. The number of proliferating fibroblasts and number and size of epithelial rests of Malassez were increased in each transgenic versus WT with double-null PDL exhibiting highest levels of proliferation and rests of Malassez at 1 month of age. Consistent with increases in PDL collagen in exon-2 deleted mice, with age, numbers of rests decreased at 12 months in this genotype. These results demonstrate for the first time a functional role of the N-propeptide in regulating collagen fiber assembly and cell behavior and suggest that SPARC and the N-propeptide of collagen I have distinct activities in regulating collagen fiber assembly and fibroblast function.

## Introduction

Collagen type I is the main structural component of the periodontal ligament (PDL), which tethers the mineralized outer layer of tooth root cementum to underlying alveolar bone [1]. Degradation and/or loss of collagen fibers within the PDL, a hallmark characteristic of periodontal disease, significantly increase the risk of alveolar bone resorption and eventual tooth loss [1–3]. Collagen I regulation and stabilization within the extracellular matrix (ECM) is critical to maintaining periodontal health. Elucidation of cellular mechanisms that control collagen incorporation and stabilization in the periodontal ECM will facilitate identification of novel targets for restoration of the periodontium and retention of alveolar bone volume and dentition, particularly in patients suffering from severe periodontal disease.

Secreted protein acidic and rich in cysteine (SPARC/osteonectin) is a matricellular protein known to bind fibrillar collagens type I and III [4]. Previous studies from our laboratory show a critical role for SPARC in maintaining collagen fiber integrity in the PDL. SPARC-null (designated ko/wt in this study) mice exhibited significant decreases in total collagen as well as decreases in thick collagen fibers in the PDL at all age points [5]. These mice also exhibited significant decreases in the force needed to extract individual teeth, suggesting that the reduced collagen content and changes in fiber morphology weakened the mechanical strength of the SPARC-null PDL when compared to wild type (WT, wt/wt) [2].

Collagen I, the most abundant component of PDL ECM, is secreted as a procollagen molecule with N- and C- terminal propeptides that undergo proteolytic cleavage prior to incorporation into the ECM [6]. The C-terminal propeptide is implicated in collagen alpha chain recognition, that takes place in the endoplasmic reticulum, and in collagen I solubility [7]. The N-propeptide of collagen I, partially encoded in exon 2 of the COL1A1 gene, has also been proposed to contribute to collagen I solubility. However, the precise functional role of the N-terminal propeptide is poorly understood. Generation of a transgenic mouse in which the gene encoding collagen ^a^1(I) was replaced with a sequence lacking exon 2 resulted in production of procollagen I lacking the portion of the N-propeptide encoded by the ^a^1(I) gene (wt/ko in this study) [8]. On first report, the lack of the ^a^1(I) N-propeptide did not appear to generate any discernable phenotypic alterations in mice [8]. Specifically, no differences in levels of dermal collagen or secretion of procollagen by dermal fibroblasts were noted in mice lacking the ^a^1(I) N-propeptide [8]. However, the dermal collagen content of SPARC-null mice carrying the exon-2 deletion (double-null, ko/ko) was significantly reduced compared to either single-transgenic alone or to WT mice. These results implicated a novel function for the N-propeptide that might work in conjunction with SPARC to facilitate collagen deposition in the ECM of dermis [9].

In this study, SPARC-null (ko/wt) mice crossed with collagen I exon-2 deleted (wt/ko) mice were used to determine whether the lack of the ^a^1(I) N-propeptide of collagen I exacerbates the lack of SPARC in terms of ECM in the PDL, a tissue with significantly higher collagen turnover compared to dermis. The current study demonstrates for the first time, an independent function of the N-propeptide as a determinant of collagen content and cellularity of the PDL, a finding not observed in skin studies. In the present study, double transgenic mice (ko/ko) were found to have a significant collagen I phenotype in PDL greater than either single transgenic alone and reflected results described in dermal tissues specifically in terms of decreases in total collagen content and increases in thin fiber morphology.

## Materials and methods

### Animal use and care

SPARC-null mice with global abrogation of SPARC expression and mice carrying the exon-2 deleted procollagen ^a^1(I) gene were crossed to generate SPARC-null/exon-2 deleted (double-null) mice as previously described [9]. Strategy to generate SPARC-null mice is described in [10] and exon-2 deleted mice in [8]. Single heterozygotes (KO/het and het/KO) were bred to obtain wild-type controls (wt/wt). Genotyping of all mice was performed by tail clip DNA extraction followed by qPCR conducted with specific forward and reverse primers utilizing the GoTaq PCR Core System I (Promega; Madison, WI). All transgenic lines were maintained on a C57Bl/6/129SV mixed background. Animals were euthanized via CO_2_ asphyxiation, followed by cervical dislocation. Mice were housed in the Ralph H. Johnson Veteran’s Administration (VA) animal facility and were supplied with a standard diet of hard chow. The described experiments in this study were conducted under an animal protocol approved by the Institutional Animal Care and Use Committee (IACUC) of the Medical University of South Carolina and the Veterans Affairs Medical Center, Charleston, South Carolina, USA.

### Histological analysis of collagen morphology and collagen area fraction

Mandibles and maxillae from single-null, double-null, and WT mice were dissected, fixed over night in zinc-formalin, and decalcified in 0.5M EDTA changed daily for 2 weeks at room temperature. Following decalcification, jaws were dehydrated in graded ethanol changes, embedded in paraffin, and sectioned (5–7μm). Sections were mounted on glass slides, rehydrated in xylene followed by incubation in a series of graded ethanol washes, washed in phosphate buffered saline containing 2% tween (PBST) for 10 minutes, then blocked in 3% donkey serum in PBST.

Picro Sirius Red (PSR) staining was performed as previously described [5]. Briefly, slides were stained in iron hematoxylin for 10 minutes followed by a running water rinse (10 min). The slides were then stained in PSR (Sigma-Aldrich; St Louis, MO) for 1 hour followed by three 5-min rinses in 1% acetic acid, dehydrated, and mounted with Clarion mounting medium and cover slips. Five mice per genotype per age group were analyzed using a minimum of 5 sections per mouse. Slides were imaged using a 40X objective on an Olympus Bx50WI microscope (Center Valley, PA) equipped with Infinity 2 Capture software (Electron Microscope Services; Hatfield, PA). Slides were visualized with polarized light under which thick collagen fibers appear bright red or red/orange and thin collagen fibers appear green/yellow in color easily differentiated against a black background [11]. Slides were also visualized and imaged under bright light, under which all collagen appears red against a yellow background. Quantitative morphometric analysis of images was conducted using Visiopharm Integrator System (VIS, version 3.2.9.0) with parameters set to measure total area of PDL, background area, total collagen area, thick collagen fiber area (red/orange), and thin collagen fiber area (green/yellow). Bright field PSR images were quantified for percent total collagen using VIS with parameters set to measure total area of red-stained collagen in the outlined region of the PDL.

Herovici histological staining was performed utilizing a kit (American MasterTech, KTHER) and following manufacturing procedure guidelines. Slides were stained with Weigert’s hematoxylin for 5 min followed by a running water rinse for 45 seconds. Herovici’s working solution was applied to slides for 2 minutes followed by a 2 minute immersion in 1% acetic acid, dehydrated for 1 min in absolute alcohol (3 changes), cleared for 1 min in histoclear (3 changes), and mounted with cytoseal wet mounting media. Slides were visualized with bright light using a 40X objective on the same scope utilized for PSR above. Newly formed collagen fibers or thin fibers were stained blue, homeostatic mature collagen or thick fibers stained red/pink, and nuclei stained black. Mean, standard deviation, and standard error of the mean (SEM) were calculated for each genotype. Values greater than two standard deviations from the mean were removed as outliers. A student’s t-test was conducted on all data and p-values<0.05 were considered significant.

### Mechanical testing of PDL

The measurement of tooth extraction force was conducted on 1-month old animals: WT, single-, and double-transgenic mandibular first molars as previously described [2]. Briefly, dissected mandibles of mice were secured to a base and a 30G wire threaded underneath the crown of the first molar was secured to an upper clamp elevating with a set rate until the point of tooth extraction. The force required for extraction was recorded with a load cell. Four mice per genotype (n = 4) were tested, using 2 technical replicates per animal (left and right mandibles from each mouse). p values < 0.05 were considered statistically significant.

### Histology and immunofluorescence of PDL cellularity

Histologically prepared mandibular and maxillary sections of 1-month old animals (n = 5) from each of the 4 genotypes were stained with hematoxylin and eosin (H&E) and visualized under bright light. Rests of Malassez were identified as multi-cellular clusters of epithelial cells within the PDL situated along the tooth cementum border [12]. Results of H&E staining were confirmed by immunofluorescence (n = 3) using a monoclonal antibody generated against mouse cytokeratin (Sigma-Aldrich, St. Louis MO). A 1:200 primary antibody concentration with overnight incubation at 4°C was followed by a 1-hour incubation with a fluorescein conjugated anti-mouse secondary antibody at a 1:200 concentration (AlexaFluor 488). Secondary-only sections were used as negative controls. A minimum of 5 sections/mouse was used for each staining protocol. Prolong anti-fade containing DAPI (Molecular Probes, Eugene, OR) was used to mount cover slips and sections were viewed on an Olympus fluorescent microscope.

Immunohistochemical staining was performed according to the following protocol. Following deparafinization and dehydration, slides were incubated in 1% hydrogen peroxide in methanol for 30 min to quench endogenous peroxidase activity. Nonspecific binding was blocked by incubating slides in 2% donkey serum in PBS for 30 min at room temperature. Slides were incubating in anti-Ki67 (abcam 15580) in a 1:500 concentration at 4°C overnight. The slides were sequentially detected with avidin-biotin-peroxidase (Thermo Scientific 32020), using 3,3’-diaminobenzidine, tetrahydrochloride (DAB, Thermo Scientific 34065) as a substrate. Nuclei were counterstained with 15% Ehrlich’s hematoxylin. Normal donkey serum was used in place of the primary antibody as a negative control. Images were quantified for the number of individual rests, for % area occupied by the rests within the PDL, and for the number of proliferating (Ki67+) cells.

Immunohistochemical multiple antigen labeling to confirm fibroblast cell type was performed by the following protocol. Slides were incubated in anti-Ki67 (abcam 15580) in a 1:500 concentration at 4°C overnight and sequentially detected with avidin-biotin-peroxidase and DAB substrate. Following PBST wash and rodent block, slides were incubated for 2 hours in mouse monoclonal anti-vimentin (abcam 8978, RV202) in a 1:50 concentration at room temperature. Slides were incubated with mouse on mouse HRP polymer (Thermo Scientific TL015QHDM) for 1 hour at room temperature, washed, developed with ImmPACT VIP substrate (Vector Laboratories SK-4605) for 10 minutes, dehydrated, cleared, and mounted using cytoseal wet mounting media. No counterstain was applied.

### Statistical analysis

The number of experimental animals used was based on previous results. A paired student’s t-test was used to calculate *p*-values within genotypes. For comparisons between genotypes with one condition a student’s t-test was used to calculate *p*-values. For comparisons between genotypes with more than one condition, ANOVA analysis followed by Tukey test was used to calculate *p*-values. For all analyses, *p* < 0.05 was considered statistically significant.

## Results

### Decreases in collagen content and fiber size of SPARC-null/exon-2 deleted PDL

**Fig 1:**
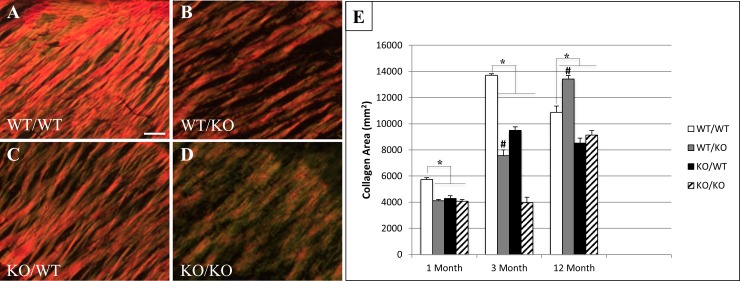
Total collagen I content is reduced in double-null PDL. Representative Picro Sirius Red (PSR) stained images of 1-month PDL from wt/wt (**A**), wt/ko (**B**), ko/wt (**C**), and ko/ko (**D**) viewed under polarized light. Images taken at 40X magnification. n = 5 mice/genotype, 5 sections/mouse. Bar in **A** = 25 μm and applies to all panels. **E.** Total collagen I content of PDL at 1-, 3-, and 12-month age points. *p<0.05 compared to wt/wt. #p<0.05 compared to ko/ko.

Previously, decreases in collagen content and fiber thickness were reported in SPARC-null (ko/wt) PDL compared to WT [5]. PSR-stained images were used to evaluate collagen content and fiber thickness in PDL of ko/ko, ko/wt, and exon-2 deleted mice (wt/ko) in comparison to wt/wt. Representative images from 1-month old mice are shown in Fig 1A–1D. Quantification of PSR images viewed under polarized light demonstrated reduced amounts of total collagen in SPARC-null (ko/wt), exon 2-deleted (wt/ko) and double-null (ko/ko) mice at 1- and 3-months of age versus WT (wt/wt) (Fig 1E). The most dramatic decrease in total collagen in double-null mice was detected at 3 months at which time the double-null PDL had significantly less collagen than either single transgenic. Whereas exon-2 deleted (wt/ko) mice exhibited reductions in total collagen at 1 and 3 months, at 12 months of age, the PDL of these mice exhibited increased amounts of total collagen versus WT mice and indicated an age-dependent effect in the N-propeptide deleted mice (Fig 1E). Likewise, double-null mice at 12 months of age demonstrated collagen content similar to that of SPARC-null mice suggesting the activity of the N-propeptide to influence collagen content was decreased at 12 months of age. Results reflecting percent total PDL collagen content were also assessed using bright field PSR images and quantification (S1 Fig).

**Fig 2:**
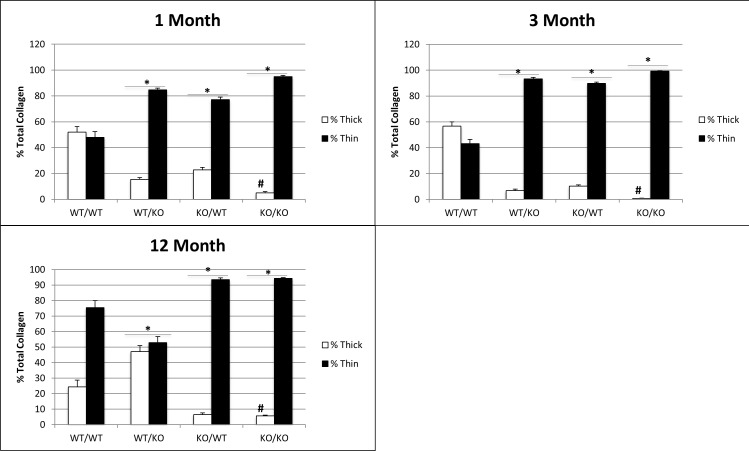
A consistent reduction in collagen I fiber size observed in double-null PDL from 1-month to 12-months of age. Collagen fiber morphology represented by thin vs. thick fibers quantified from PSR histology for three age points. n = 5 mice/genotype, 5 sections/mouse. *p<0.05 compared to wt/wt counterparts, #p<0.05 compared to single transgenics.

Morphological analysis of PSR-stained images revealed thinner collagen fibers in each of the transgenic mice at 1- and 3-months of age versus that in WT PDL (Fig 2). At 3 months of age, double-null PDL had significantly less thick collagen fiber content than either of the single transgenics. At 12 months of age, mice lacking exon 2 demonstrated significantly more thick fiber content in the PDL than WT, SPARC-null, or double null PDL. The persistence of thin fiber content in SPARC-null PDL was also reflected in the thin fiber content of double null mice at 12 months of age (Fig 2). Hence, similar to collagen content, effects of the N-propeptide in regulating collagen fiber assembly appear to shift with age whereas the lack of SPARC in double-null mice appears to dominate collagen fiber morphology at 12 months.

**Fig 3:**
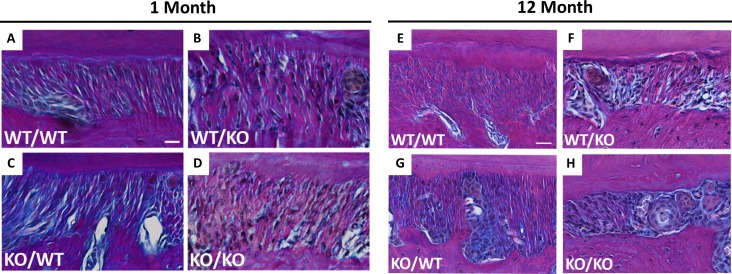
Collagen fiber morphology altered in double-null PDL at 1- and 12-month age points. Representative images of Herovici stained PDL from wt/wt (**A**&**E**), wt/ko (**B**&**F**), ko/wt (**C**&**G**), and ko/ko (**D**&**H**) shown at 1- and 12-months of age respectively. Red/pink stain indicates thicker collagen fibers; blue stain indicates thinner collagen fibers, nuclei stain black. Images captured at 40X and oriented with alveolar bone on bottom, PDL center, and tooth cementum on top. Bar in **A**&**E** = 25 μm and applies to all panels. n = 3 mice/genotype, 5 section/mouse.

Herovici stained 1- and 12-month PDL sections reveal distinct staining patterns of collagen fibers in each genotype (Fig 3). Thicker collagen fibers stain red/pink with Herovici stain whereas thinner fibers appear blue. At 1 month of age, wt/wt PDL stained with Herovici’s stain showed the presence of both red and blue fibers (Fig 3A) whereas in wt/wt PDL at 12 months of age, red fibers appeared to dominate the sections (Fig 3E), suggesting maturation of collagen fibers with age. Interestingly, an apparent increase in red fibers in 1-month wt/ko over that of wt/wt PDL was noted (Fig 3B vs. 3A). Overall apparent decreases in stain intensity are visible in ko/ko 1 month PDL indicative of overall reduction in collagen content (Fig 3D). At 12 months of age (Fig 3E–3H), differences between transgenic mice were not as significant as the 1-month time point. Interestingly, the predominant red staining of collagen fibers evident at 1 month in wt/ko PDL appears to be reduced at 12 months of age (Fig 3F). Double-transgenic ko/ko 12 month PDL displays loss of red-stained fibers when compared to single transgenics; resulting in a PDL consisting primarily of blue-stained collagen fibers (Fig 3H).

### Mechanical strength of PDL

**Fig 4:**
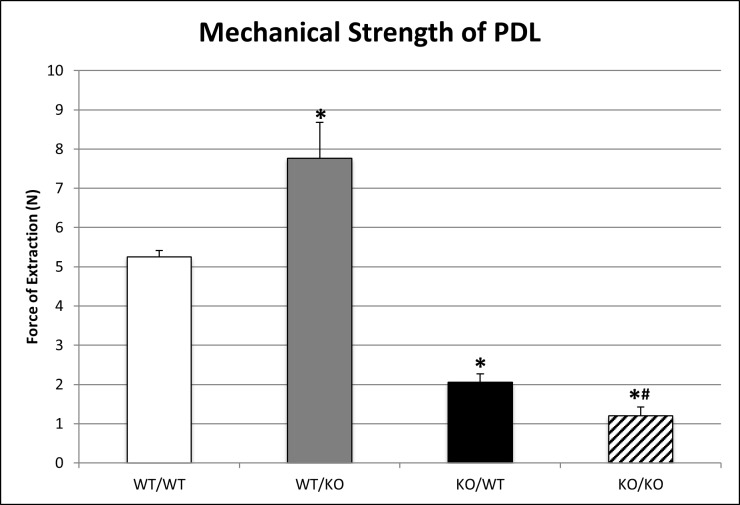
Mechanical force of tooth extraction varies among transgenic PDL. Quantification of n = 4 mice, 2 replicates/mouse. *p<0.05 compared to wt/wt, #p<0.05 compared to single transgenics.

Reductions in total collagen content and fiber morphology have been reported to correlate with decreases in mechanical strength of SPARC-null PDL [2]. Measurements of the force required to extract teeth from each genotype at 1 month of age are shown in Fig 4. Consistent with previous reports, the force required to extract SPARC-null (ko/wt) teeth was significantly decreased compared to WT and exon-2 deleted (wt/ko) teeth [13]. Double-null (ko/ko) PDL exhibited further decreases in mechanical strength compared to both WT and SPARC-null (ko/wt) PDL (Fig 4). Unexpectedly, exon-2 deleted (wt/ko) teeth required greater forces to extract teeth compared to WT (wt/wt) despite reduced amounts of total collagen and thick collagen at 1 month of age as measured by PSR stain (Figs 1 & 2).

### Increases in fibroblast proliferation in SPARC-null/exon-2 deleted PDL

**Fig 5:**
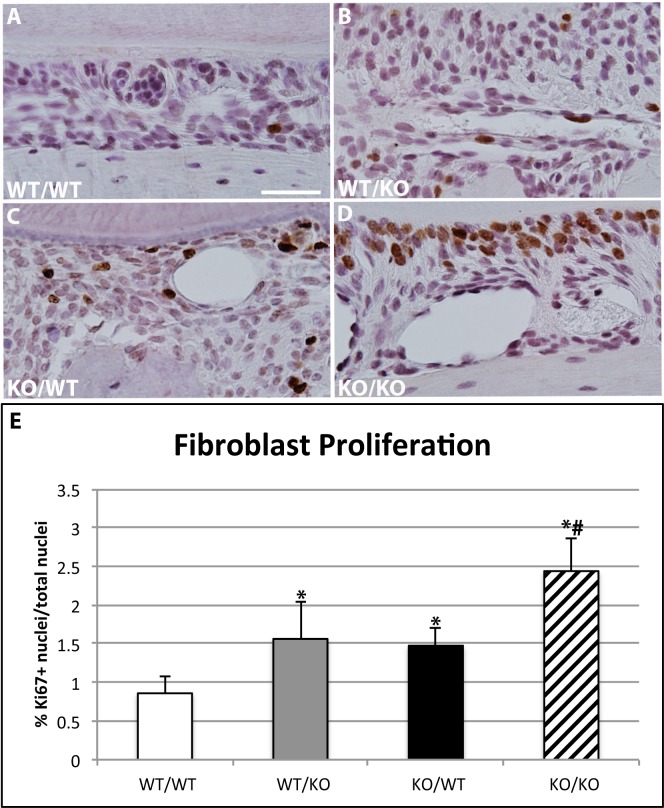
Ki67 expression is increased in single- and double-transgenic murine PDL. Representative images of Ki67 immunohistochemistry localized in sections of PDL from wt/wt (**A**), wt/ko (**B**), ko/wt (**C**), and ko/ko (**D**) 1-month mice. All images are oriented with tooth on top, PDL center, and alveolar bone on bottom; 40X magnification. Bar in **A** = 100 μm and applies to all panels. **E.** Quantification of n = 5 mice/genotype, 5 sections/mouse. *p<0.05 compared to wt/wt, #p<0.05 compared to single transgenics.

Fibroblasts are the primary cell type that synthesize and secrete collagen in the PDL [14]. To detect whether transgenic mice exhibited differences in fibroblast proliferation immunohistochemistry, using Ki67 as a marker of proliferating cells, was performed. Representative images from each genotype are shown in Fig 5A–5D. Quantification of Ki67 positive cells demonstrated a marked increase in proliferating cells in SPARC-null (ko/wt) and exon-2 deleted (wt/ko) PDL over that of WT PDL at 1 month (Fig 5E). Increases in Ki67 positive cells were observed in 1-month double-null PDL compared to single transgenics or WT PDL. Double antigen labeling using Ki67 and vimentin supported that the predominant cell type displaying increases in proliferating cells were PDL fibroblasts (S2 Fig).

### Increases in epithelial rests of malassez in SPARC-null/exon-2 deleted PDL

**Fig 6:**
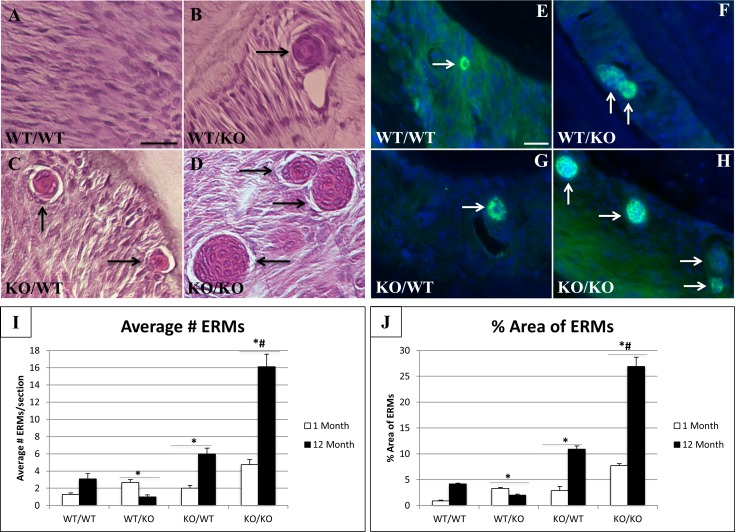
Number and size of epithelial rests of Malassez (ERMs) varies in murine periodontal ligament (PDL). Representative sections from 1 month wt/wt (**A**), wt/ko (**B**), ko/wt (**C**), and ko/ko (**D**) PDL stained with H&E. Black arrows indicate multi-cellular ERMs. Bar in **A** = 25 μm and applies to all panels. Representative images from 1 month PDL stained for cytokeratin immunoreactivity (green); wt/wt (**E)**, wt/ko (**F**), ko/wt (**G**), ko/ko (**H**). Nuclei stained blue (DAPI). White arrows indicate multi-cellular rests of Malassez expressing cytokeratin. Images are representative of n = 3 mice/genotype. **I**. Quantification of average number of rests present in sampled PDL. **J**. Percent of PDL area occupied by rests. n = 5. *p<0.05 compared to wt/wt, #p<0.05 compared to single transgenics.

H&E staining of transgenic PDL revealed an increase in the number of rests of Malassez in SPARC-null/exon-2 deleted (ko/ko) versus WT (Fig 6A–6D) as well as increased size of ERMs (determined by %PDL occupied by ERMs) in 1-month SPARC-null/exon 2-deleted PDL (ko/ko) compared to single transgenics and WT (Fig 6J). Cytokeratin is present in the periodontium during tooth and PDL development in the enamel organ, junctional epithelium, and in rests of Malassez [15]. In adults, the only remnants of cytokeratin expression in the PDL are found in the rests of Malassez. Immunofluorescence using a pan-cytokeratin antibody confirmed significant increases in number of ERMs/PDL section (Fig 6E–6H). At 12 months of age, double-null PDL displays expansion of ERMs and PDL area occupied by these rests in comparison to less dramatic increases found in WT and SPARC-null PDL (Fig 6I and 6J). Interestingly, the number and area of the ERMs decreased in PDL of exon-2 deleted mice at 12 months in comparison to both 1-month values and WT values at 12 months (Fig 6I).

## Discussion

This study evaluated whether the N-propeptide of collagen I influences ECM assembly and fibroblast proliferation in the PDL and if loss of the N-propeptide in combination with loss of SPARC expression further exacerbates the defects in PDL collagen characteristic of SPARC-null PDL. Collagen content and fiber morphology in the PDL of exon 2-deleted and double-null mice were significantly compromised, with double-null mice demonstrating the most significant loss of collagen at 3 months of age. The force required to extract teeth at 1 month of age was also lowest in double-null mice versus that of either single transgenic or WT mice, indicating that the loss of collagen content and integrity directly correlated to a decrease in mechanical stability of the PDL. Fibroblast proliferation and the number of rests of Malassez were increased in SPARC-null and exon 2-deleted PDL with the greatest elevations observed again in double-null PDL compared to WT PDL. Interestingly, exon-2 deleted mice demonstrated the highest level of force required for molar extraction at 1 month of age despite reductions in collagen and thick fiber content reported by PSR. Differences in PDL collagen characterized herein for exon-2 deleted mice are the first phenotypic alterations reported for these mice and thus demonstrate functional significance for the N-propeptide in collagen fiber formation in vivo.

The function of the N-propeptide in collagen biology is not well defined [6]. Mutations found in patients that either reduce or prevent processing of the N-propeptide from collagen I give rise to malformed collagen fibrils and weaker tissue strength. Dermatosporaxis is a disease associated with inefficiency in processing of the N-propeptide of collagen I due to reduced or abrogated enzymatic activity of the N-propeptide proteinase, ADAMTS2/3 and 14, that gives rise to fragile skin among other pathologies [16]. Clearly, retention of the N-propeptide of collagen I is detrimental to collagen fiber assembly and tissue formation and is presumably mediated by interference of the retained N-propeptide in collagen fibrils. Studies carried out in cultured cells suggested that the N-propeptide might act as a feedback inhibitor of collagen production [17]. As such, mice lacking the N-propeptide were anticipated to demonstrate increases in collagen content brought about by the lack of this inhibitory function. The exon 2-deleted mice were originally generated to test this hypothesis.

Bornstein et al. reported that exon 2-deleted mice lacked an overt collagen phenotype characterized primarily at 2 months of age in skin, although there was some embryonic lethality on specific backgrounds [8]. Similarly, our laboratory reported a lack of phenotypic differences in adult skin of exon-2 deleted mice [9]. However, when coupled with a lack of SPARC expression, robust differences in dermal collagen content and morphology were found in SPARC-null/exon-2 deleted mice over and above that of SPARC-null mice, perhaps suggesting a cooperative role of SPARC and N-propeptide. In contrast to skin, at 1 month of age the PDL of exon-2 deleted mice displayed decreased total collagen and increases in thin fibers. Interestingly, this collagen phenotype appeared to recover in the exon-2 deleted PDL by the 12-month age point. Perhaps, a compensatory mechanism supported by other collagens present in the PDL of exon-2 deleted mice mediates recovery. In addition to type I, type III collagen is highly expressed by human gingival and PDL fibroblasts [18]. The N-propeptide of procollagen III is synthesized in a very comparable pattern to that of procollagen I and has similarities in sequence and structure to the N-propeptide of collagen I [19]. Hence, functional redundancy of the N-propeptide of procollagen III might compensate for the lack of the type I N-propeptide in the exon-2 deleted mouse and explain the collagen I phenotypic recovery observed in these mice at later time points. We also speculate that the high rate of collagen turnover in the PDL affords the opportunity to discern functional activities of the N-propeptide that might be more subtle in tissues with slower collagen turnover such as the skin.

Few published reports characterizing collagen fiber analysis by Herovici stain are available. Reynolds et al. reported that collagen fibers, when treated with collagenase, undergo changes in Herovici stain color from red to blue. In addition, tissues with higher levels of thin collagen fibers, such as embryonic and those with healing wounds, demonstrate increases in blue stained collagen fibers over red fibers [20]. In our hands, differences in blue and red-stained fibers are evident in 1- and 12-month murine PDL. Particularly, the exon-2 deleted 1 month PDL (Fig 3B) that displays an apparent increase in red-stained fibers, compared to WT, that can be observed weaving into tooth and bone. Herovici staining might provide an improved method for visualizing thin collagen fibers, particularly in the PDL, versus PSR because of the inherent strong PSR signal from surrounding collagen rich tissues (bone and tooth). In contrast to other soft tissues, not surrounded by collagen-rich mineralized tissues, the distinction between thick and thin collagen fibers in the PDL can be challenging by PSR histology. An increase in thick collagen fibers in exon-2 deleted mice visualized with Herovici stain would be consistent with increases in mechanical strength observed in these mice at a month of age.

At 1 month of age, the mechanical strength of the double transgenic SPARC-null/exon-2 deleted PDL was significantly decreased compared to all other genotypes as evidenced by the decrease in force required for molar extraction. This result is in line with the decreased collagen content and fiber thickness observed histologically. The exon-2 deleted mice were the only transgenic line studied that displayed increased mechanical strength of PDL compared to WT, a surprising result based on the reduced amount of collagen I observed at 1 month versus WT mice. A possible explanation is that subtle differences in collagen structure might exist in the exon-2 deleted PDL that account for increases in strength of the ECM in the absence of overt differences in collagen fiber morphology or content. For example, changes in ECM structural properties originating from alterations to collagen cross-linking could contribute to the increased mechanical strength of the PDL observed in exon-2 deleted mice. Future experiments to understand how ECM composition might alter mechanical properties of the PDL should be enlightening.

Fibroblast proliferation, as measured by Ki67 positive cells, was increased in the PDL of both single-transgenic mice. The increase in proliferation was further exaggerated in double-null PDL compared to single transgenics and WT. Fibroblasts are known to bind and respond to growth factors sequestered in the matrix, particularly during wound healing [21]. In an ECM environment characterized by decreased collagen content such as that of the transgenic mice, factors normally sequestered, for example fibroblast growth factor-2 (FGF-2), might be present in higher concentrations as active, soluble factors that stimulate fibroblast proliferation versus WT PDL. Perhaps the increase in fibroblast proliferation might represent a compensatory mechanism to replenish the lack of collagen content in double-null PDL, for example.

Epithelial Rests of Malassez (ERMs) were also increased in the PDL of transgenic mice. ERMs are cell clusters from Hertwig’s epithelial rooth sheath that remain in the PDL after root formation is complete. ERMs are known to proliferate and increase in size under conditions of PDL stress, such as orthodontic tooth movement, but their functional role in the ECM is poorly understood [22]. ERMs are capable of undergoing epithelial-mesenchymal transition and differentiate into various cell types [23]. These cell rests are speculated to play a role in regeneration and repair of the periodontium. The increase in number and area of these rests observed in single-transgenic mice and further exaggerated in double-transgenic mice could serve as an indicator of a stressed and weakened PDL. As each of these transgenic mice displayed decreases in thick collagen and increases in thin collagen, one might speculate that each also has increased tooth movement during mastication. The increases in tooth movement resulting from a weaker PDL might explain the increase in ERMs observed in transgenic mice when compared to WT. ERMs are also known to proliferate with age in homeostatic murine PDL [24]. Cell adhesion and proliferation potentially contribute to epithelial cell rest proliferation and growth within the PDL matrix. However, Yan et al. reported no differences in lens epithelial cell proliferation in the absence of SPARC compared to WT *in vivo* [25]. Since the absence of SPARC does not affect epithelial proliferation globally, we do not highlight epithelial cell expansion as a main factor altering the ERM-matrix structure. The increased presence of ERMs in 12-month WT PDL is attributed to aging PDL turnover and remodeling. The significant increase in SPARC-null rests at 12 months compared to WT correlates with the decrease in total collagen content (Fig 1E) and a decrease in the amount of thick fibers present in 12-month PDL (Fig 2). We hypothesize the failure of SPARC-null PDL to recover collagen content at 12-months indicates a stressful/weakened ECM resulting in further ERM proliferation. Interestingly the recovery of the collagen phenotype observed in exon-2 deleted PDL at 12 months (Fig 1E & Fig 2) is reflected in a decrease in the number and area of ERMs occupying the PDL at this age. Double-null PDL at 12 months, was shown to have substantial decreases in collagen content and morphology likely resulting in a robust proliferation of both number and size of ERMs (Fig 6I and 6J).

We conclude that the absence of SPARC expression combined with a lack of the N-terminal propeptide exon 2 altered collagen type I morphology, cellularity, and mechanical stability of the PDL. The mechanisms behind the activities of both SPARC and the N-propeptide of collagen I need further exploration and could provide insight into collagen assembly and ECM remodeling. Further studies will highlight potential targets in the pathway of collagen assembly and regeneration, applicable to tissues suffering from collagen deficiency or following an injury disease state such as periodontal disease.

## Supporting information

S1 Fig Percent total collagen I content is reduced in double-null PDL at all age points. Representative bright field images of Picro Sirius Red stained 1-month PDL from wt/wt (**A**), wt/ko (**B**), ko/wt (**C**), ko/ko (**D**). Images taken at 40X magnification. Images are oriented with alveolar bone on bottom, PDL center, and tooth cementum on top. n = 5 mice, 5 sections/mouse. Bar in **A** = 25 μm and applies to all panels. Method of quantification is shown in **E** followed by graphical representation in **F** of PDL at 1-, 3-, and 12-month age points. *p<0.05 compared to wt/wt. #<0.05 compared to ko/ko.
(TIF)

S2 Fig Ki67 and vimentin double antigen labeling supports increases in fibroblast proliferation in transgenic PDL. Representative images of Ki67 and vimentin immunohistochemistry localized in sections of PDL from wt/wt (**A**), wt/ko (**B**), ko/wt (**C**), ko/ko (**D**) 1-month old mice. Ki67+ cells stained brown and vimentin+ cells stained purple. All images are oriented with alveolar bone on bottom, PDL center, and tooth cementum on top. Images taken at 40X magnification. Bar in **A** = 50 μm and applies to all panels. Black arrows indicate representative cells positive for both Ki67 and vimentin. White arrows indicate representative Ki67 positive, vimentin negative cells. Black arrowheads indicate representative Ki67 negative, vimentin positive cells.
(TIF)
