# Supplementary figures and images for: SPARC and the N-propeptide of collagen I influence fibroblast proliferation and collagen assembly in the periodontal ligament
Source: PLoS One. 2017 Feb 28;12(2):e0173209. doi: 10.1371/journal.pone.0173209 (PMC5330531; doi:10.1371/journal.pone.0173209)

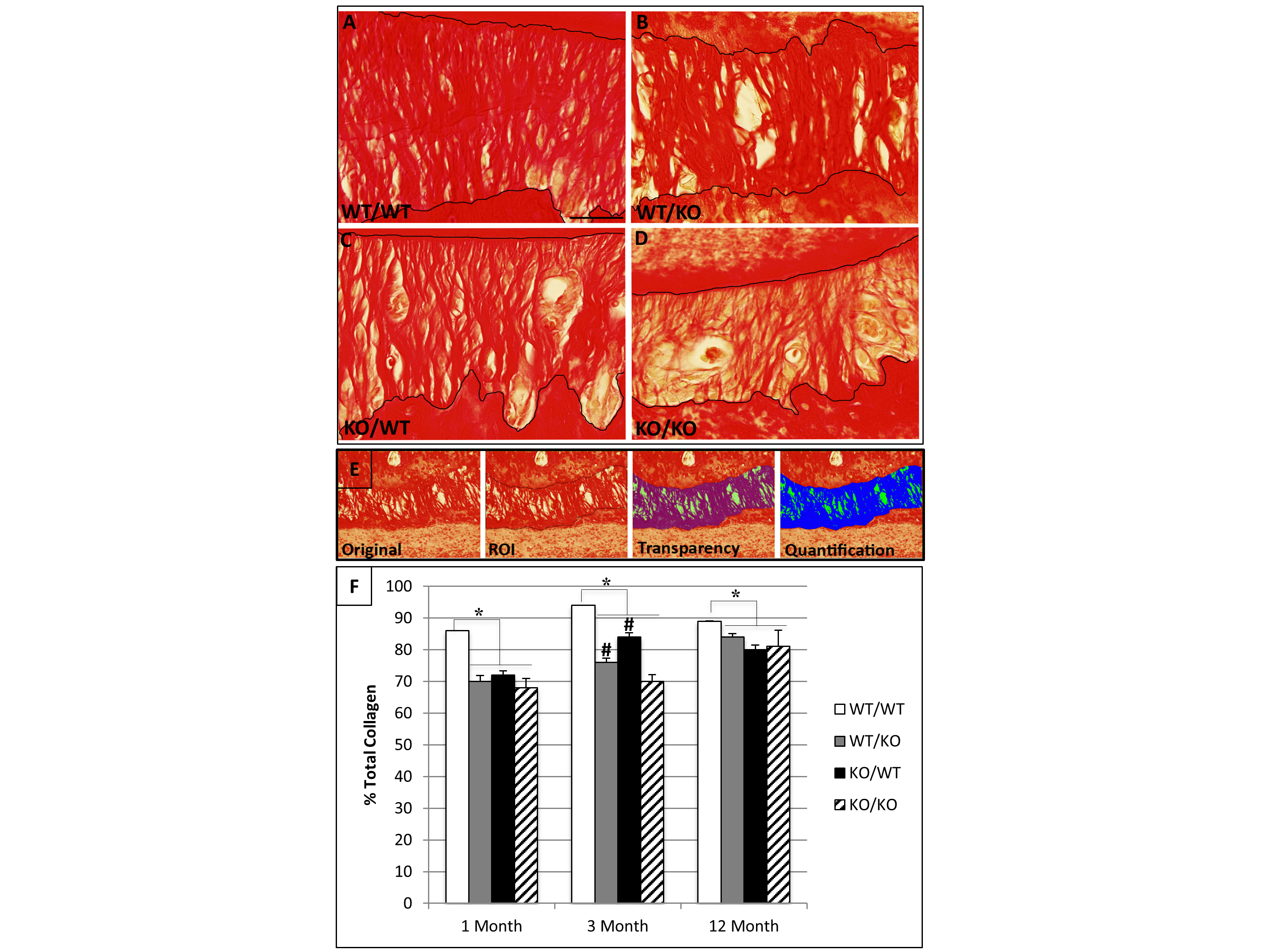

Supplement: S1 Fig — Representative bright field images of Picro Sirius Red stained 1-month PDL from wt/wt (A), wt/ko (B), ko/wt (C), ko/ko (D). Images taken at 40X magnification. Images are oriented with alveolar bone on bottom, PDL center, and tooth cementum on top. n = 5 mice, 5 sections/mouse. Bar in A = 25 μm and applies to all panels. Method of quantification is shown in E followed by graphical representation in F of PDL at 1-, 3-, and 12-month age points. *p<0.05 compared to wt/wt. #<0.05 compared to ko/ko. (TIF) [file pone.0173209.s001.TIF]

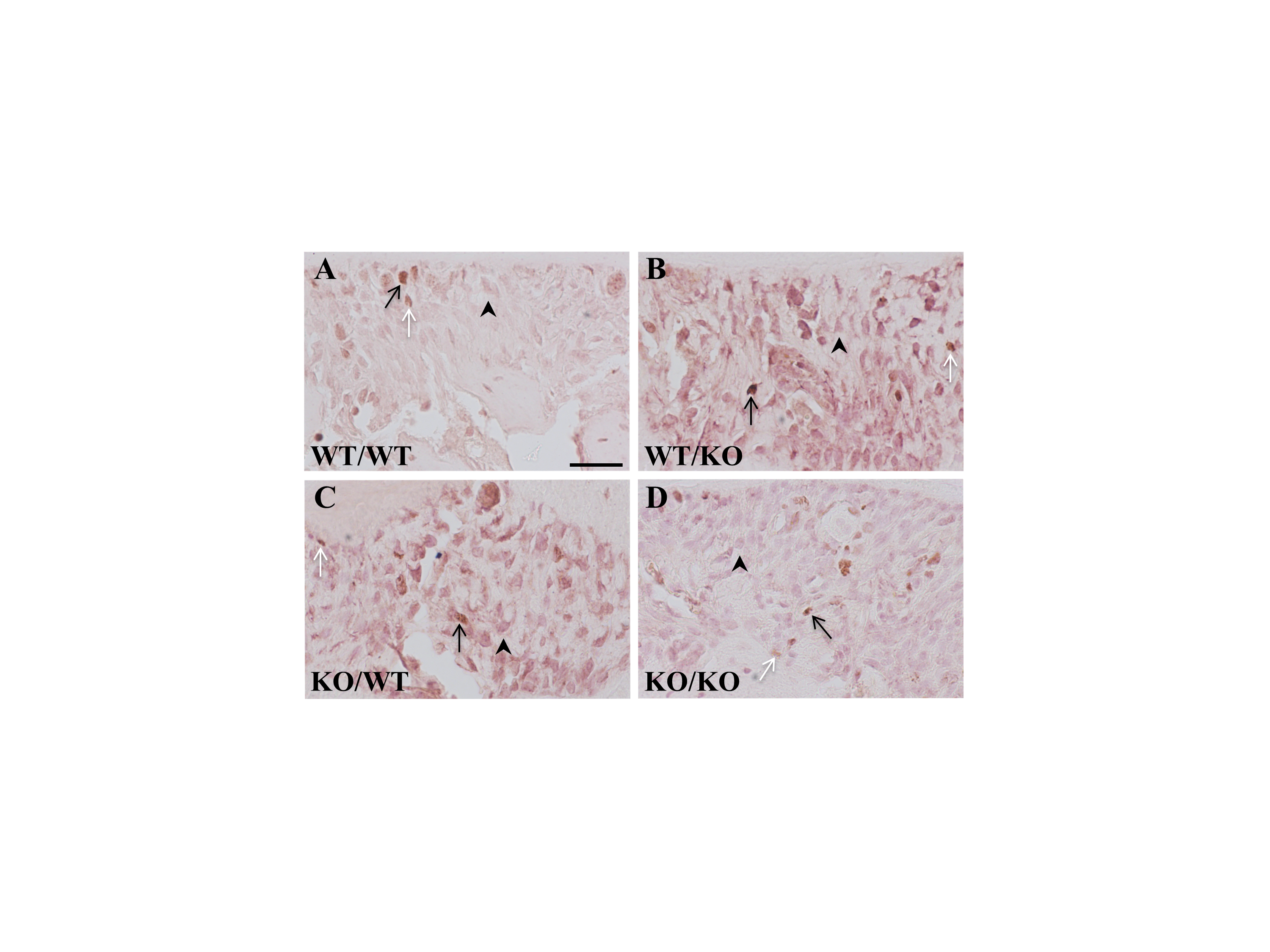

Supplement: S2 Fig — Representative images of Ki67 and vimentin immunohistochemistry localized in sections of PDL from wt/wt (A), wt/ko (B), ko/wt (C), ko/ko (D) 1-month old mice. Ki67+ cells stained brown and vimentin+ cells stained purple. All images are oriented with alveolar bone on bottom, PDL center, and tooth cementum on top. Images taken at 40X magnification. Bar in A = 50 μm and applies to all panels. Black arrows indicate representative cells positive for both Ki67 and vimentin. White arrows indicate representative Ki67 positive, vimentin negative cells. Black arrowheads indicate representative Ki67 negative, vimentin positive cells. (TIF) [file pone.0173209.s002.TIF]
